# ROS-Scavenging Nanomaterials to Treat Periodontitis

**DOI:** 10.3389/fchem.2020.595530

**Published:** 2020-11-04

**Authors:** Lihua Sui, Jianling Wang, Zuoxiu Xiao, Yuqi Yang, Zhichun Yang, Kelong Ai

**Affiliations:** ^1^Department of Pharmacology, Xiangya School of Pharmaceutical Sciences, Central South University, Changsha, China; ^2^Hunan Provincial Key Laboratory of Cardiovascular Research, Central South University, Changsha, China

**Keywords:** periodontitis, ROS, antioxidants, nanomaterials, polydopamine

## Abstract

The incidence of periodontitis is very high, and up to 45–50% of people are suffering from periodontitis. Periodontitis is caused by pathogens that invade teeth-supporting tissues such as gingiva, periodontal ligament, and alveolar bone. Pathogens trigger host immune responses characterized by the overproduction of reactive oxygen species (ROS). The development of effective ROS scavengers through nanotechnology has been emerging as a promising strategy for the treatment of periodontitis. Nanomaterial-based antioxidants can effectively scavenge ROS, prevent ROS-mediated tissue damage, and relieve inflammation in periodontitis. This mini-review focuses on the generation of ROS in periodontitis and its molecular mechanism of destroying periodontal tissue. Meanwhile, we summarize the research progress of ROS-scavenging nanomaterials in the treatment of periodontitis and discuss the challenges and prospects of its application.

## Introduction

Periodontitis is a common inflammatory disease with an overall prevalence of 45–50%, and 11.2% of the world's population are affected by severe periodontitis (Kassebaum et al., 2014; Sanz et al., 2020). The host immune response triggered by the pathogens colonized on the subgingival plaque can lead to overproduction of ROS, causing oxidative stress and damage to the gingiva, alveolar bone, periodontal ligament, and other teeth-supporting tissues. As a result, the affected teeth become mobility and eventually fall off (Kinane et al., 2011; Tóthová and Celec, 2017; Wang et al., 2017). Furthermore, periodontitis also influences the occurrence and progression of systemic health. Studies have confirmed that excessive ROS in periodontitis led to systemic arterial endothelial dysfunction, which is a potential risk factor for atherosclerosis (Yamamoto et al., 2016). Administration of exogenous antioxidants can scavenge excessive ROS from periodontal tissues and reduce inflammation. However, most conventional antioxidants exist shortcomings including poor aqueous dispersibility, low stability, and short duration of action. Hence, developing feasible antioxidant materials is of great significance to treat periodontitis.

In recent years, ROS-scavenging nanomaterials have exhibited promising prospects in the field of periodontitis treatment. Three advantages of nanomaterials in this field can be highlighted: (i) delivery of drugs to specific therapeutic sites; (ii) high biocompatibility and low toxicity *in vivo*; and (iii) capability of promoting periodontal tissue regeneration (Kahraman et al., 2016; Bao et al., 2018; Higuchi et al., 2019). Based on this, the review focuses on the generation and pathogenic mechanism of ROS in periodontitis, and then summarize the applications of ROS-scavenging nanomaterials.

## ROS in the Pathogenesis of Periodontitis

ROS refer to the general term for oxygen free radicals and peroxides, which mainly include hydroxyl radicals (·OH), superoxide anions (·${\text{O}}_{2}^{-}$), singlet oxygen (^1^O_2_), and hydrogen peroxide (H_2_O_2_). There is a balance between the production of ROS and antioxidant defenses under physiological conditions. The respiratory chain and most enzymatic reactions can lead to the release of ROS, which is essential for biological processes such as intracellular signal transduction and gene expression regulation (Finkel, 2011). However, once the overproduction of ROS beyond the scavenging ability of the endogenous antioxidant defense system, it will cause oxidative stress, and destroy tissues *via* DNA and protein damage, lipid peroxidation, and enzymatic oxidation (Sies, 1997; Chapple and Matthews, 2007).

**Figure 1:**
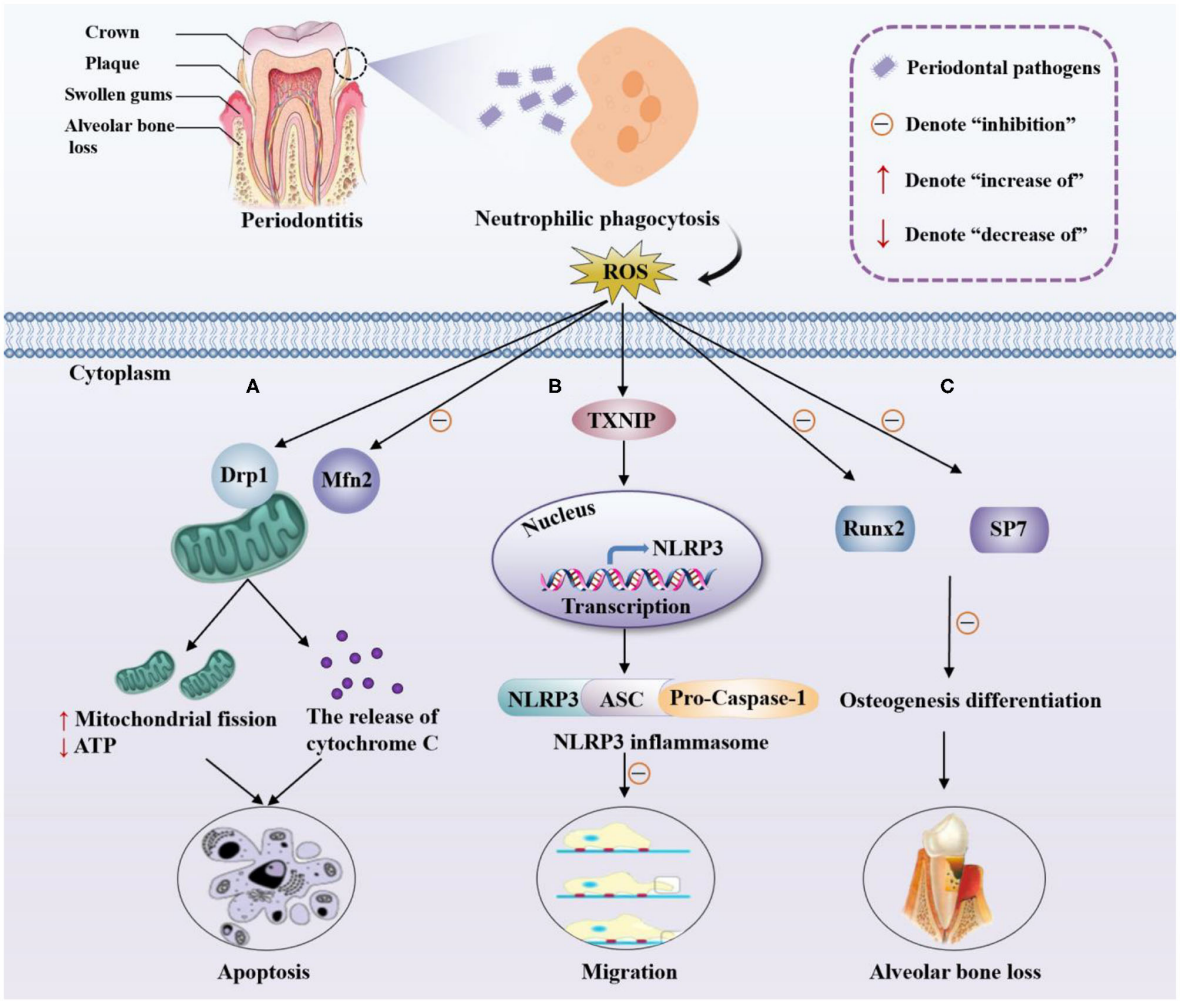
Pathomechanism of periodontitis and ROS. Pathogens in dental plaque trigger the recruitment and activation of neutrophils, producing a large amount of ROS through phagocytosis. **(A)** ROS promote mitochondrial fission via the upregulation of Drp1 and downregulation of Mfn2 which is a mitochondrial fusion protein, reduce the production of ATP, and increase the release of cytochrome C, inducing the apoptosis of PDLSCs. **(B)** ROS trigger the combination of TXNIP and NLRP3, leading to the upregulation of the transcription of NLRP3, thereby activating the NLRP3 inflammasome and ultimately inhibiting the migration of PDLFs. **(C)** ROS inhibits the expression of osteogenic markers Runx2 and SP7, inhibiting the osteogenic differentiation of periodontal membrane stem cells, which cause alveolar bone loss.

ROS play an important role in the pathomechanism of periodontitis. The pathogens colonized on the dental plaque are mainly Gram-negative anaerobic bacteria that can trigger the recruitment and activation of neutrophils. Subsequently, neutrophils produce a series of antibacterial factors during the phagocytosis of periodontal pathogens and release excessive ROS through the NADPH oxidase pathway (Ramesh et al., 2016; Misawa et al., 2019). Although ROS produced by immune cells have antibacterial effects, excessive ROS can cause oxidative stress, interfere the cell cycle progression, and induce apoptosis of gingival fibroblasts. Besides, ROS also act as intracellular signaling transduction molecules to promote the formation of osteoclasts, leading to alveolar bone resorption and damage of periodontal tissues (Kanzaki et al., 2017). Current researches on the involvement of ROS in the pathogenesis of periodontitis mainly focus on apoptosis of periodontal ligament stem cells, migration of periodontal ligament fibroblasts, and alveolar bone resorption (alveolar bone loss) (Figure 1). For instance, He et al. confirmed that ROS are critical in the apoptosis of periodontal ligament stem cells (PDLSCs) (He et al., 2018). ROS significantly increased the expression of dynamin-related protein 1 (Drp1), the primary regulator in mitochondrial fission, leading to the mitochondrial dysfunction, including abnormal mitochondrial membrane potential and reduced ATP level, which ultimately causes apoptosis of PDLSCs (Figure 1A).

ROS can also damage the periodontal ligament by inhibiting the migration of periodontal ligament fibroblasts. Lian et al. demonstrated that LPS treatment of periodontal ligament fibroblasts (PDLFs) could lead to the overproduction of ROS, triggering the combination of thioredoxin interacting protein (TXNIP) and NOD-Like receptor protein 3 (NLRP3), and inducing NLRP3 inflammasome formation. NLRP3, ASC (apoptosis-associated speck-like protein containing a CARD), and Pro-Caspase-1 (the effector cysteine protease caspase 1) are the three components of NLRP3 inflammasome. The activation of NLRP3 inflammasome can cause the pathogenicity in cells beyond the classical inflammatory response. This series of changes inhibited the migration of mPDLFs and ultimately caused damage to the periodontal ligament (Lian et al., 2018) (Figure 1B).

Another important pathological process of periodontitis is alveolar bone resorption that could result in the gradual loosening and loss of teeth. With self-renewal ability, multipotency, and high biocompatibility, human PDLSCs (hPDLSCs) have become a source of periodontal tissue regeneration and exhibited the ability to repair alveolar bone defects in periodontitis (Wen et al., 2019). Substantial evidence has confirmed that the osteogenic differentiation of hPDLSCs was impaired in periodontitis patients, suggesting that ROS contribute to osteoclast activation and result in pathological bone destruction. The role of forkhead box O1 (FoxO1), a member of the FoxO family, in oxidative stress and osteogenesis of hPDLSCs was reported (Huang and Chen, 2019). Their research showed that the antioxidant effect of FoxO1 could protect hPDLSCs from oxidative damage and promote osteogenesis. Specifically, overexpression of FoxO1 reduced ROS accumulation, decreased malondialdehyde (MDA) levels, and increased the expression of the osteogenic markers runt related transcription factor 2 (Runx2) and Sp7 transcription factor (SP7) (Figure 1C).

## ROS-Scavenging Nanomaterials in Periodontitis

### Elimination of ROS by Drug-Loaded Nanoparticles

Antioxidants can attenuate the irreversible destruction of teeth-supporting tissues caused by excessive ROS. Conventional antioxidants, such as genistein, melatonin, and resveratrol, cannot be applied locally due to their poor aqueous dispersibility. Alternatively, they are hard to permeate into periodontal tissue and may cause side effects (Kara et al., 2013; Tamaki et al., 2014; Bhattarai et al., 2017). These problems can be efficiently solved by drug-loaded nanoparticles. Murgia et al. have developed a novel buccal drug delivery system to treat oral diseases related to oxidative stress topically (Murgia et al., 2019). Curcumin (CUR) is a natural polyphenolic compound that has been studied for its antioxidant effect (Menon and Sudheer, 2007). Due to its chemical instability and water insolubility, the application of CUR is limited. For this reason, they prepared bioadhesive buccal tablets containing CUR-loaded nanostructured lipid carriers (CUR-NLC) and demonstrated that the ability of NLC to promote the penetration of CUR through the lipophilic domains of the mucosal membrane. In addition, the advantages of high drug loading, specific site release, and prolonged drug action have also made nanomaterials very promising for periodontitis (Goyal et al., 2014). Nevertheless, nanocarriers loaded with antioxidant drugs are still in their initial state, and only a few examples have been used for the treatment of periodontitis. It is worth noting that the few examples showed great potential of antioxidant drug-loaded nanocarriers on the treatment of periodontitis (Table 1).

**Table 1:** Applications of ROS-scavenging nanomaterials to treat periodontitis.

**Nanomaterials**	**Polymers**	**Research mode**	**Quantitaive information**	**Advantages**	**References**
**Drug-loaded nanoparticles**					
Coenzyme Q10-loaded nanomicelles	Kolliphor® P 407	*In vitro* and *in vivo*	GI values decreased from 2.04 to 0.88, PI decreased from 2.06 to 0.80, and PPD also shallowed by about 41%	Improved drug stability and significantly ameliorate periodontal parameters	Shaheen et al., 2020
Biopolymer-silica composites loaded with *L. divaricata* Cav. extract	Chi and CMC	*In vitro*	The degree of incorporation of the extract for Chi-CMC-SiO_2_ composites was 44.36%	Presented a high activity of simil SOD and capacity to eliminate free radicals	Alvarez Echazú et al., 2018
**ROS-responsive nanoparticles**					
Novel redox injectable gel	PMNT-PEG-PMNT triblock copolymer	*In vitro* and *in vivo*	The degree of bone loss of Pg and RIG@Pg rats was 0.479 ± 0.038 and 0.341 ± 0.035 mm, respectively	Recovered gingival blood flow and inhibited alveolar bone loss	Saita et al., 2016
Polydopamine nanoparticles	Polydopamine	*In vitro* and *in vivo*	PDA NPs with a dosage of 0.2 mg/site can decrease ROS-related fluorescence intensity	Exhibited biodegradable and admirable anti-inflammatory activity	Bao et al., 2018

*Chi, Chitosan; CMC, carboxymethylcellulose; PMNT-PEG-PMNT, poly[4-(2,2,6,6-tetramethylpieridine-N-oxyl)aminomethylsyrene]-b-poly(-ethylene glycol)-b-poly[4-(2,2,6,6-tetramethylpiperidine-N-oxyl)aminomethylstyrene]; GI, gingival index; PI, plaque index; PPD, periodontal pocket depth; Pg, porphyromonas gingivalis*.

Recently, Shaheen et al. has demonstrated that nanomaterials loaded with antioxidants could be administrated locally in periodontal pockets to effectively treat periodontitis (Shaheen et al., 2020). They prepared a micellar nanocarrier containing coenzyme Q10 (Q10) by a modified nanoprecipitation method and then evaluated the treatment effects of this innovative system in moderate periodontitis. Q10 is an antioxidant produced by the body itself, but gingival cells of patients with periodontitis are seriously deficient in Q10. Exogenous supplementation of Q10 is necessary for the medical treatment of periodontitis patients. Loading Q10 into ultra-small size nanoparticles could improve its aqueous dispersibility and bioavailability. In their study, Q10 was formulated in nano-micelles (NMQ10) that was incorporated *in situ* gelling systems, followed by injection into the periodontal pockets of periodontitis patients. The results showed that the NMQ10 was able to penetrate into the required site well. Periodontitis patients who received the administration of NMQ10 obtained a significant therapeutic effect, with significantly reduced oxidative stress markers and improved periodontal evaluation parameters.

Also, incorporating plant extracts into nanocomposites can contribute to the development of effective therapeutic drugs to scavenge ROS from periodontal tissue. The nanocomposite loaded with the *Larrea divaricata* Cav. aqueous extract has been introduced (Alvarez Echazú et al., 2018). *Larrea divaricata* Cav. is a South American plant that shows antioxidant activity suitable for treating periodontal diseases, and its aqueous extract contains nordihydroguaiaretic acid and other ingredients that can scavenge ROS. Incorporation of this aqueous extract into nanocomposites could achieve an effective release of the extract at a specific treatment site. Chitosan (Chi) and carboxymethylcellulose (CMC) are mucoadhesive biopolymers that are usually used to deliver drugs to oral tissues. As a bioactive material that promotes the proliferation and differentiation of osteoblasts, silica (SiO_2_) was also considered to be a candidate compound for the treatment of periodontitis. Collectively, the incorporation of the *Larrea divaricata* Cav. aqueous extract into Chi-CMC-SiO_2_ nanoparticle composites exhibited good biodegradability and antioxidant activity. It could effectively remove stable free radical DPPH and present superoxide dismutase-like activity that prevents excessive ROS production.

### Elimination of ROS by Nanoparticles

In addition to ROS scavenging, nanoparticles that respond to ROS themselves are also used for antioxidant therapy in periodontitis (Table 1). Current researches related to periodontitis mainly use two types of ROS-responsive nanomaterials. One is the redox injectable gel that is formed by the disintegration of nano-assembled flower micelles, and the other is antioxidant nanoparticles with nano-enzyme properties.

ROS-scavenging nanomaterials with prolonged retention time around the lesional area would be widely used in the treatment of periodontitis. A novel redox injectable gel (RIG) that can effectively scavenge ROS and inhibit alveolar bone resorption has been designed (Saita et al., 2016). RIG was prepared from a triblock copolymer with nitroxide radicals as a specific ROS scavenger. Nitroxide radical compounds have superoxide dismutase (SOD)-like activity that can scavenge ROS such as ·${\text{O}}_{2}^{-}$ and ·OH (Samuni et al., 1988). The triblock copolymer forms polyion complexes with anionic poly (acrylic acid) (PAAc) to form a flower-like micelle (ca. 79 nm), which exhibits *in situ* thermo-irreversible gelation under physiological conditions at 37°C. The gelation contributes to long-term retention of the drug after injection at a special site and exerting a strong antioxidant effect (Pua et al., 2013). Saita's results showed that RIG could be effectively retained in the mouth and recover gingival blood flow after administration in periodontal pockets of periodontitis rats. Meanwhile, RIG could significantly relieve the lipid peroxidation damage caused by ROS and inhibit alveolar bone resorption by suppressing differentiation of osteoclast precursor cells to osteoclast.

Different from the mechanism of ROS removal by RIG, nanoparticles with antioxidative enzyme-like activity have been studied to relieve ROS-triggered inflammatory reaction. Bao et al. utilized polydopamine nanoparticles (PDA NPs) as an intelligent multi-ROS scavenger to develop a biodegradable and efficient antioxidant defense platform, which exhibited ideal outcomes (Bao et al., 2018). Polydopamine (PDA) is a major pigment of naturally occurring melanin (eumelanin) that displays excellent biocompatibility and its derivatives have been widely used in biomedical research (Simon and Peles, 2010). PDA-based materials possess broad development prospects in scavenging multi-ROS because their chemical structure incorporates many functional groups such as catechol, amine, and imine, which shows powerful reducing capability (Liu et al., 2014). Bao et al. have synthesized PDA NPs via a classical StÃber method and their research has proved that PDA NPs have high biological safety and admirable removal capacity toward various ROS. Furthermore, PDA NPs could efficiently decrease the activity of some pro-inflammatory cytokines including tumor necrosis factor α (TNF-α) and interleukins (IL-1β), suppressing local inflammation after administration of PDA NPs in periodontitis mouse.

## Summary and Outlook

In summary, this mini-review has elucidated the production of ROS and its pathogenic mechanism in periodontitis, and discussed three aspects of the pathogenic mechanism of ROS from the apoptosis of periodontal ligament stem cells, the migration of periodontal ligament fibroblasts and alveolar bone resorption. Overproduction of ROS causes the release of inflammatory factors to trigger a series of biological events such as the degradation of periodontal fibers, alveolar bone resorption, and ultimately tooth loosening and loss. Based on this, we summarized the nanomaterial-based ROS scavengers currently used for the antioxidant treatment including nanomaterials loaded with antioxidants and ROS-responsive nanoparticles. These ROS-scavenging nanomaterials have demonstrated great potential in the treatment of periodontitis, ascribable to their good biocompatibility, high permeability, and prolonged retention time in periodontal tissues.

Periodontitis is a highly prevalent health problem worldwide that negatively affects the life quality of many populations. Excessive ROS produced by the host immune response worsen the conditions and the topical application of antioxidants can enhance the treatment of periodontitis. Disappointingly, most traditional antioxidants have unsatisfactory results in the treatment of local inflammatory diseases. To some extent, this is because of non-specific distribution, poor permeability of specific tissues, and low retention in the lesion (Li et al., 2018). Thanks to the advantages of nanotechnology, the ROS-scavenging nanomaterials can be carefully designed according to the specific biological environment to improve its biological stability and accumulation in periodontal tissues, to give full play to its antioxidant treatment of periodontitis. ROS-scavenging nanomaterials promise to be remarkable candidates for the treatment of periodontitis to solve the vital problems of pain, recurrent attacks, and high cost. Although ROS-scavenging nanomaterials have made considerable progress in the treatment of periodontitis, this emerging field is still at its early stage. There are still many scientific issues and current challenges to be solved: (1) Since the high humidity of the oral environment and its secretions will dilute the drug, multiple applications are required to achieve a continuous action. Therefore, in terms of mucosal adhesion and release of active substances, the study of bioactive carriers is necessary (Alvarez Echazú et al., 2018). (2) ROS play a crucial role under physiological conditions, so it is vital to determine the correct dosage of ROS-scavenging nanomaterials to guide intracellular ROS to achieve therapeutic effects rather than pathological effects. (3) In order to further accelerate its clinical translation, the long-term biological effects of ROS-scavenging nanomaterials should be studied in depth in animals and humans (Yang and Chen, 2019).

In the future, more research should focus on loading drugs with stronger antioxidants or more stable properties into nanomaterials and developing targeted nanomaterials with multiple responses (e.g., temperature and pH) for controlled release of drugs at indicated points. Additionally, ROS-scavenging nanomaterials with high biocompatibility, biodegradability, and drug loading capacity are urgently needed. Although there is relatively little research on ROS-scavenging nanomaterials that can be safely used for a long time and can sustainable release of bioactive compounds, it has also attracted great interest in the field of periodontitis treatment and has an extremely broad application prospect.

## Author Contributions

LS, JW, KA, and ZY wrote the manuscript. ZX and YY revised the manuscript. All authors contributed to the article and approved the submitted version.

## Conflict of Interest

The authors declare that the research was conducted in the absence of any commercial or financial relationships that could be construed as a potential conflict of interest.

## References

[B1] Alvarez Echazú M. I. Olivetti C. E. Peralta I. Alonso M. R. Anesini C. Perez C. J.. (2018). Development of pH-responsive biopolymer-silica composites loaded with *Larrea divaricata* Cav. extract with antioxidant activity. Colloid. Surf. B Biointerfaces 169, 82–91. 10.1016/j.colsurfb.2018.05.015 29751344

[B2] Bao X. Zhao J. Sun J. (2018). Polydopamine nanoparticles as efficient scavengers for reactive oxygen species in periodontal disease. ACS Nano 12, 8882–8892. 10.1021/acsnano.8b04022 30028940

[B3] Bhattarai G. Poudel S. B. Kook S. H. Lee J. C. (2017). Anti-inflammatory, anti-osteoclastic, and antioxidant activities of genistein protect against alveolar bone loss and periodontal tissue degradation in a mouse model of periodontitis. J. Biomed. Mater. Res. A 105, 2510–2521. 10.1002/jbm.a.36109 28509410

[B4] Chapple I. L. Matthews J. B. (2007). The role of reactive oxygen and antioxidant species in periodontal tissue destruction. Periodontol 43, 160–232. 10.1111/j.1600-0757.2006.00178.x 17214840

[B5] Finkel T. (2011). Signal transduction by reactive oxygen species. J. Cell Biol. 194, 7–15. 10.1083/jcb.201102095 21746850 PMC3135394

[B6] Goyal G. Garg T. Rath G. Goyal A. K. (2014). Current nanotechnological strategies for an effective delivery of drugs in treatment of periodontal disease. Crit. Rev. Ther. Drug Carrier Syst. 31, 89–119. 10.1615/critrevtherdrugcarriersyst.2014008117 24940625

[B7] He Y. Gan X. Zhang L. Liu B. Zhu Z. Li T.. (2018). CoCl(2) induces apoptosis via a ROS-dependent pathway and Drp1-mediated mitochondria fission in periodontal ligament stem cells. Am. J. Physiol. Cell Physiol. 315, C389–C397. 10.1152/ajpcell.00248.2017 29768044

[B8] Higuchi J. Fortunato G. Wozniak B. Chodara A. Domaschke S. Meczyńska-Wielgosz S.. (2019). Polymer membranes sonocoated and electrosprayed with nano-hydroxyapatite for periodontal tissues regeneration. Nanomaterials. 9:111625. 10.3390/nano9111625 31731775 PMC6915502

[B9] Huang X. Chen H. (2019). FoxO1 overexpression ameliorates TNF-α-induced oxidative damage and promotes osteogenesis of human periodontal ligament stem cells via antioxidant defense activation. Stem Cells Int. 2019:2120453. 10.1155/2019/2120453 31781234 PMC6875375

[B10] Kahraman E. Özhan G. Özsoy Y. Güngör S. (2016). Polymeric micellar nanocarriers of benzoyl peroxide as potential follicular targeting approach for acne treatment. Colloid. Surf. B Biointerfaces 146, 692–699. 10.1016/j.colsurfb.2016.07.029 27434156

[B11] Kanzaki H. Wada S. Narimiya T. Yamaguchi Y. Katsumata Y. Itohiya K.. (2017). Pathways that regulate ROS scavenging enzymes, and their role in defense against tissue destruction in periodontitis. Front. Physiol. 8:351. 10.3389/fphys.2017.00351 28611683 PMC5447763

[B12] Kara A. Akman S. Ozkanlar S. Tozoglu U. Kalkan Y. Canakci C. F.. (2013). Immune modulatory and antioxidant effects of melatonin in experimental periodontitis in rats. Free Radic. Biol. Med. 55, 21–26. 10.1016/j.freeradbiomed.2012.11.002 23146767

[B13] Kassebaum N. J. Bernabé E. Dahiya M. Bhandari B. Murray C. J. Marcenes W. (2014). Global burden of severe periodontitis in 1990-2010: a systematic review and meta-regression. J. Dent. Res. 93, 1045–1053. 10.1177/0022034514552491 25261053 PMC4293771

[B14] Kinane D. F. Preshaw P. M. Loos B. G. Working Group 2 of Seventh European Workshop on Periodontology. (2011). Host-response: understanding the cellular and molecular mechanisms of host-microbial interactions—Consensus of the Seventh European Workshop on Periodontology. J. Clin. Periodontol. 38, 44–48. 10.1111/j.1600-051X.2010.01682.x 21323703

[B15] Li L. Guo J. Wang Y. Xiong X. Tao H. Li J.. (2018). A broad-spectrum ROS-eliminating material for prevention of inflammation and drug-induced organ toxicity. Adv Sci. 5:781. 10.1002/advs.201800781 30356945 PMC6193162

[B16] Lian D. Dai L. Xie Z. Zhou X. Liu X. Zhang Y.. (2018). Periodontal ligament fibroblasts migration injury via ROS/TXNIP/Nlrp3 inflammasome pathway with *Porphyromonas gingivalis* lipopolysaccharide. Mol. Immunol. 103, 209–219. 10.1016/j.molimm.2018.10.001 30312877

[B17] Liu Y. Ai K. Lu L. (2014). Polydopamine and its derivative materials: synthesis and promising applications in energy, environmental, and biomedical fields. Chem. Rev. 114, 5057–5115. 10.1021/cr400407a 24517847

[B18] Menon V. P. Sudheer A. R. (2007). Antioxidant and anti-inflammatory properties of curcumin. Adv. Exp. Med. Biol. 595, 105–125. 10.1007/978-0-387-46401-5_3 17569207

[B19] Misawa M. Y. O. Silvério Ruiz K. G. Nociti F. H. Jr. Albiero M. L. Saito M. T. Nóbrega Stipp R.. (2019). Periodontal ligament-derived mesenchymal stem cells modulate neutrophil responses via paracrine mechanisms. J. Periodontol. 90, 747–755. 10.1002/jper.18-0220 30644104

[B20] Murgia D. Angellotti G. D'Agostino F. (2019). Bioadhesive matrix tablets loaded with lipophilic nanoparticles as vehicles for drugs for periodontitis treatment: development and characterization. Polymers 11:111801. 10.3390/polym11111801 31684081 PMC6918209

[B21] Pua M. L. Yoshitomi T. Chonpathompikunlert P. Hirayama A. Nagasaki Y. (2013). Redox-active injectable gel using thermo-responsive nanoscale polyion complex flower micelle for noninvasive treatment of local inflammation. J. Contr. Rel. 172, 914–920. 10.1016/j.jconrel.2013.10.009 24157475

[B22] Ramesh A. Varghese S. S. Doraiswamy J. N. Malaiappan S. (2016). Herbs as an antioxidant arsenal for periodontal diseases. J. Intercult. Ethnopharmacol. 5, 92–96. 10.5455/jice.20160122065556 27069730 PMC4805154

[B23] Saita M. Kaneko J. Sato T. Takahashi S. S. Wada-Takahashi S. Kawamata R.. (2016). Novel antioxidative nanotherapeutics in a rat periodontitis model: reactive oxygen species scavenging by redox injectable gel suppresses alveolar bone resorption. Biomaterials 76, 292–301. 10.1016/j.biomaterials.2015.10.077 26559357

[B24] Samuni A. Krishna C. M. Riesz P. Finkelstein E. Russo A. (1988). A novel metal-free low molecular weight superoxide dismutase mimic. J. Biol. Chem. 263, 17921–17924. 2848018

[B25] Sanz M. Del Castillo A. M. Jepsen S. Gonzalez-Juanatey J. R. D'Aiuto F. Bouchard P.. (2020). Periodontitis and cardiovascular diseases. Consensus report. Glob. Heart 15, 1–1. 10.5334/gh.400 32489774 PMC7218770

[B26] Shaheen M. A. Elmeadawy S. H. Bazeed F. B. Anees M. M. Saleh N. M. (2020). Innovative coenzyme Q(10)-loaded nanoformulation as an adjunct approach for the management of moderate periodontitis: preparation, evaluation, and clinical study. Drug Deliv. Transl. Res. 10, 548–564. 10.1007/s13346-019-00698-z 31953677

[B27] Sies H. (1997). Oxidative stress: oxidants and antioxidants. Exp. Physiol. 82, 291–295. 10.1113/expphysiol.1997.sp004024 9129943

[B28] Simon J. D. Peles D. N. (2010). The red and the black. Acc. Chem. Res. 43, 1452–1460. 10.1021/ar100079y 20734991

[B29] Tamaki N. Cristina Orihuela-Campos R. Inagaki Y. Fukui M. Nagata T. Ito H. O. (2014). Resveratrol improves oxidative stress and prevents the progression of periodontitis via the activation of the Sirt1/AMPK and the Nrf2/antioxidant defense pathways in a rat periodontitis model. Free Radic. Biol. Med. 75, 222–229. 10.1016/j.freeradbiomed.2014.07.034 25091897

[B30] Tóthová L. Celec P. (2017). Oxidative stress and antioxidants in the diagnosis and therapy of periodontitis. Front. Physiol. 8:1055. 10.3389/fphys.2017.01055 29311982 PMC5735291

[B31] Wang Y. Andrukhov O. Rausch-Fan X. (2017). Oxidative stress and antioxidant system in periodontitis. Front. Physiol. 8:910. 10.3389/fphys.2017.00910 29180965 PMC5693842

[B32] Wen Y. Yang H. Wu J. Wang A. Chen X. Hu S.. (2019). COL4A2 in the tissue-specific extracellular matrix plays important role on osteogenic differentiation of periodontal ligament stem cells. Theranostics 9, 4265–4286. 10.7150/thno.35914 31285761 PMC6599665

[B33] Yamamoto Y. Saito T. Feng G. G. Li J. Yasuda Y. Kazaoka Y.. (2016). Intermittent local periodontal inflammation causes endothelial dysfunction of the systemic artery via increased levels of hydrogen peroxide concomitantly with overexpression of superoxide dismutase. Int. J. Cardiol. 222, 901–907. 10.1016/j.ijcard.2016.08.099 27526356

[B34] Yang B. Chen Y. (2019). Reactive oxygen species (ROS)-based nanomedicine. Chem. Rev. 119, 4881–4985. 10.1021/acs.chemrev.8b00626 30973011

